# Characterization of a Decapentapletic Gene (*AccDpp*) from *Apis cerana cerana* and Its Possible Involvement in Development and Response to Oxidative Stress

**DOI:** 10.1371/journal.pone.0149117

**Published:** 2016-02-16

**Authors:** Guilin Li, Hang Zhao, Hongfang Wang, Xulei Guo, Xingqi Guo, Qinghua Sun, Baohua Xu

**Affiliations:** 1 State Key Laboratory of Crop Biology, College of Life Sciences, Shandong Agricultural University, Taian, Shandong, 271018, P. R. China; 2 College of Animal Science and Technology, Shandong Agricultural University, Taian, Shandong, 271018, P. R. China; Sichuan University, CHINA

## Abstract

To tolerate many acute and chronic oxidative stress-producing agents that exist in the environment, organisms have evolved many classes of signal transduction pathways, including the transforming growth factor β (TGFβ) signal pathway. Decapentapletic gene (*Dpp*) belongs to the TGFβ superfamily, and studies on *Dpp* have mainly focused on its role in the regulation of development. No study has investigated the response of *Dpp* to oxidative pressure in any organism, including *Apis cerana cerana* (*A*. *cerana cerana*). In this study, we identified a *Dpp* gene from *A*. *cerana cerana* named *AccDpp*. The 5΄ flanking region of *AccDpp* had many transcription factor binding sites that relevant to development and stress response. *AccDpp* was expressed at all stages of *A*. *cerana cerana*, with its highest expression in 15-day worker bees. The mRNA level of *AccDpp* was higher in the poison gland and midgut than other tissues. Furthermore, the transcription of *AccDpp* could be repressed by 4°C and UV, but induced by other treatments, according to our qRT-PCR analysis. It is worth noting that the expression level of *AccDpp* protein was increased after a certain time when *A*. *cerana cerana* was subjected to all simulative oxidative stresses, a finding that was not completely consistent with the result from qRT-PCR. It is interesting that recombinant *AccDpp* restrained the growth of *Escherichia coli*, a function that might account for the role of the antimicrobial peptides of *AccDpp*. In conclusion, these results provide evidence that *AccDpp* might be implicated in the regulation of development and the response of oxidative pressure. The findings may lay a theoretical foundation for further genetic studies of *Dpp*.

## Introduction

*Apis cerana cerana* (*A*. *cerana cerana*) is a well-known subspecies of oriental bees. Compared with *Apis mellifera* (*A*. *mellifera*), *A*. *cerana cerana* has a strong resistance to mites, acute sense of smell, and can forage the nectar and pollen of wide range of flowers, including wild plants. These advantages are irreplaceable by *A*. *Mellifera* [1,2,3]. However, recently, excessive uses of pesticides and the existence pollutants, climate change with extreme heat and cold, ultraviolet radiation, and heavy metals in the environment, which can lead to the generation of reactive oxygen species (ROS), cause serious harm to the survival of honeybees [4, 5, 6, 7].

ROS homeostasis and signalling are essential to the organisms, but their exact function remains a mystery. Hydrogen peroxide (H_2_O_2_), hydroxyl radical (HO•), and superoxide anion (O^2−^), are generated endogenously or exogenously by ROS. A low concentration of ROS is essential to the organism, and accumulating evidence has suggested that ROS can serve as pivotal signalling molecules to participate in the regulation of various cellular functions, including cell growth, proliferation, survival and the immune response [8, 9]. However, excess ROS result in various disease states, including cancer, aging, diabetes, and neurodegeneration, and are implicated in the damage of macromolecules, such as lipids, protein, and nucleic acids. Generally, oxidative stress occurs when antioxidant defence mechanisms are compromised or antioxidant protection is overwhelmed by a high level of ROS. Redox-sensitive signalling proteins can be modified by oxidative stress, which leads to aberrant cell signalling [10]. As signalling molecules, ROS may be connected to many signal pathways by targeting transduction or specific metabolic cellular components, which may execute and initiate the program of cell apoptotic death. Transforming growth factor β (TGFβ) signal transduction can also be activated by ROS.

The TGFβ superfamily was first discovered by Robert [11] and successfully extracted in human blood by Assoian [12]. TGFβ superfamily members include TGFβs, activins, bone morphogenetic protein (BMP), inhibins, and growth differentiation factor (GDF). There are many subtypes in the TGFβ superfamily. In mammals, the subtypes mainly include TGFβ1, TGFβ2, and TGFβ3, which are expressed in various tissues and have different expression levels [13]. TGFβ plays an essential role in signaling pathways that control metazoan cell growth, differentiation, and participates in the formation of tissues and organs, as well as in the immune response of the body [14, 15].

Previous studies have also indicated that TGFβ signalling could mediate ROS production and control redox [16]. The increase in ROS contributes to TGFβ-induced cell apoptosis in cirrhotic hepatocytes [17], while the inhibition of ROS lowers the susceptibility of the cell to TGFβ-induced apoptosis. High levels of TGFβ, IL6, and IL1 can induce a ROS-mediated response to DNA damage [18]. Chen et al. (2012) proposed that the signalling pathway involved in ROS could increase TGFβ expression, resulting in increased ROS production and promoting TGFβ-dependent fibrogenesis [19]. TGFβ elicited cell apoptosis in mouse foetal hepatocytes via an oxidative process [20]. The expression of catalase and MnSOD can be regulated by TGFβ in airway smooth muscle cells, leading to the change ROS levels in the body [21]. Michaeloudes et al. (2011) suggested that TGFβ upregulated the level of *Nox4*, possibly generating H_2_O_2_ and ultimately contributing to the increase of ROS. Renal autoregulation could be impaired by TGFβ through the generation of ROS [22]. ROS can be induced by TGFβ and then activate *p38* [23, 24, 25]. TGFβ participates in mediating transglutaminase 2 activation in the oxidative stress response, causing protein aggregation [26]. However, in *A*. *cerana cerana*, the TGFβ signalling pathway has not been studied.

*Dpp* (decapentapletic gene), similar to *BMP2* and *BMP4* of vertebrates, is a member of the TGFβ superfamily in insects and is a secreted molecule. It was first found and studied more clearly in *Drosophila melanogaster* (*D*. *melanogaster*). In Drosophila embryonic development, *Dpp* is one type of segment polarity gene that belongs to the group of zygotic genes. Dpp is a morphogen in the process of insect development and guides cell growth, differentiation and senescence in a dosage-dependent manner [27, 28, 29]. Many previous genetic analyses have demonstrated that *Dpp* also played a crucial role in many developmental events through positional information in the intercellular signalling pathway [30, 31]. Ninov et al. (2010) found that Dpp signalling pathways can directly regulate cell motility and retraction [32]. There are many genes in the signalling pathway of Dpp, such as *Dpp*, *Put*, *Tkv*, *Mad*, *Med*, *Shn*, and *Brk*, among which *Dpp* is located the most upstream. The Dpp signalling pathway has been implicated in many developmental processes and can both activate and repress gene transcription [33]. Studies of *Dpp* have mainly focused on its role in growth and development. Although *Dpp* is a member of the TGFβ superfamily, whether *Dpp* is related to ROS remain unknown.

*A*. *cerana cerana* plays a critical role in the development of honey industry and maintains the ecological balance. Though its genome information had been uncovered in 2015 [34], its was not be released. In addition, to date, only 195 mRNA sequences of *A*. *cerana cerana* have been submitted in the NCBI database. Thus, it is essential to obtain more information concerning gene expression for the study of the function and biological mechanisms of Chinese bees. To our knowledge, the role of *Dpp* in *A*. *cerana cerana* has not been studied. In this paper, we isolated and characterized the *Dpp* gene from *A*. *cerana cerana* and detected its expression profile in different tissues, at different development stages, and under various oxidative stresses at the mRNA and protein levels. So far, this is the first report concerning the relationship between the *Dpp* gene and oxidative stress.

## Materials and Methods

### Experimental insects and various treatments

The insects (*A*. *cerana cerana*) used in this work were reared in the artificial beehives of Shandong Agricultural University (Taian, China). In general, each colony has one queen to lay eggs, which has completed mating and will stay in the hive all the time, unless swarming or flying fled. Honey bees of different developmental stages were classified based on the criteria of previous reports [35]. The egg (Eg), one-day to seven-day larvae (L1-L7), pre-pupal phase pupae (Po), pupae (white-eyed (Pw), pink-eyed (Pp), brown-eyed (Pb) and dark-eyed (Pd) pupae)), and 1-day worker bees (A1) were collected directly from the hive, while adult honey bees (15-day worker bees (A15), and 30-day worker bees (A30)) were collected at the entrance of the hive by marking 1-day worker bees with paint 15 and 30 days earlier. The 15-day worker bees were divided into ten groups (n = 40/group) and kept at 34°C under standard conditions as described by Alaux et al. (2010) [36]. Each group was treated with various stress conditions (S1 Table), which could be involved in oxidative stress [4, 5, 6, 7], and the control groups (untreated 15-day worker bees) were incubated at 34°C and fed with normal food. Bees that were injected with phosphate buffered saline (PBS) (0.5 ul/worker) were the injection controls of group injected with H_2_O_2_. Methomyl, Vitamin C (VC), HgCl_2_ and CdCl_2_ were dissolved in water, and acaricide, cyhalothrin and paraquat were diluted by water. The honeybees in the above experiments were collected at the indicated time. To analyse tissue-specific expression, different tissues of the 15-day worker bees, including the leg, wing, muscle, midgut, haemolymph, rectum, poison gland, honey sac, antennae and epidermis, were dissected on ice. All of the specimens were flash-frozen in liquid nitrogen and stored at -70°C until they were used. Each experiment was performed in triplicate.

### Extraction of total RNA, synthesis of cDNA and genomic DNA preparation

Total RNA from *A*. *cerana cerana* was extracted and cDNA was synthesized using TRIzol reagent (Invitrogen, Carlsbad, CA, USA) and an EasyScript First-Strand cDNA Synthesis SuperMix (TransGen Biotech, Beijing, China), respectively, as per the manufacturers’ protocol. For expression profile analysis of *AccDpp* at different development and under different types of abiotic stresses, whole honeybee was used to extract RNA, and for the analysis of the expression patterns of *AccDpp* at different development, the RNA was extracted from different tissues. The extraction of genomic DNA was performed according to the instructions offered by the EasyPure Genomic DNA Extraction Kit (TransGen Biotech, Beijing, China).

### Primers and amplification conditions

The primer pairs used in this study are listed in S2 Table and were synthesized by Sangon Biotechnological Company (Shanghai, China). All of the polymerase chain reaction (PCR) amplification procedures are listed in S3 Table.

### Cloning of the full-length cDNA, 5′-flanking region, and genomic sequence of *AccDpp*

Acquisition of the *AccDpp* full-length cDNA, 5′-flanking region, and genomic sequence was carried out as described by Chen et al. (2015) [37].

### Bioinformatics analysis

The MatInspector database (http://www.cbrc.jp/research/db/TFSEARCH.html) was used to predict the putative transcription factor binding sites (TFBs) of the *AccDpp* promoter. The GC content of the gene was predicted by the DNASTAR program (version 7.01). NCBI servers (http://blast.ncbi.nlm.nih.gov/Blast.cgi) were used to select the homologous sequence of AccDpp and to predict the conserved domain of Dpp from different species. DNAMAN version 5.22 (Lynnon Biosoft, Quebec, Canada) and the ProtParam tool (http://www.expasy.ch/tools/protparam.html) were used to determine the physical and chemical properties of *AccDpp*. Molecular Evolutionary Genetics Analysis (MEGA version 4.1) was chosen to generate the phylogenetic tree. The prediction of antimicrobial peptides and signal peptide of AccDpp was performed using the antimicrobial peptide database and Signalp 4.1 Server, separately. The online software SWISS-MODEL was used to build the possible three-dimensional structure of AccDpp, and SPDBV version 4.1 was chosen to analyze the three-dimensional structure of AccDpp.

### Fluorescent real-time quantitative PCR

Fluorescent real-time quantitative PCR (qRT-PCR) was carried out according to the protocol of Zhang et al. (2013) to check the mRNA expression profile of *AccDpp* [38]. The expression of *AccDpp* was normalized by *β-actin* (GenBank Accession No. HM-640276), which is stably expressed [39, 40, 41, 42]. Untreated 15-day worker bees were used as controls.

### Protein expression, purification and preparation of anti-*AccDpp*

The stop codon and signal peptide-less open reading frame (ORF) of *AccDpp* with the KpnI and SacI restriction sites were cloned into the expression vector pET-30a(+) (Novagen, Madison, WI) and was transformed into *Transetta* (DE3) chemically competent cells (*Escherichia coli*, TransGen Biotech, Beijing, China). The induction and purification of recombinant AccDpp were performed based on previous reports [38]. The preparation of antibodies were performed according to the procedure of Meng et al. (2014) with some modification [43]. In brief, the target protein was separated by 12% SDS-PAGE. The SDS-PAGE albumin glue that contained the target protein was cut and ground with moderate benzylpenicillin sodium for injection (Lukang Pharmaceutical, Jining, China) and sodium chloride injection (0.9%) (Cisen Pharmaceutical, Jining, China). The ground sample was used to inject white mice (Taibang, Taian, China).

### Western blot analysis

The total protein of *A*. *cerana cerana* was extracted and quantified according to the protocol provided by a tissue protein extraction kit (ComWin Biotech, Beijing, China) and a total protein assay kit (using a standard BCA method; ComWin Biotech, Beijing, China), respectively. After equal amounts of the protein of each sample were separated by 12% SDS-PAGE, they were electrotransferred onto a PVDF membrane (ComWin Biotech, Beijing, China) using the wet transfer method. Then, membrane rinsed with 10 mL of TBST buffer solution containing 0.5 g of Difco^™^ Skim Milk (Solarbio, Beijing, China). The primary antibodies (anti-AccDpp polyclonal antibody; 1:100 dilution) were used to incubate the membrane at 4°C overnight. After rinsing in TBST three times, the secondary antibodies (peroxidase-conjugated goat anti-mouse immunoglobulin G; Jingguo Changsheng Biotechnology, Beijing, China) at a dilution of 1:2000 (v/v) were used to probe the membrane. Finally, the membrane was washed with TBST. The results of antigen-antibody binding were detected using the SuperSignal^™^ West Dura Extended Duration Substrate (Thermo Fisher Scientific, Shanghai, China).

### Disc diffusion assay

*Escherichia coli* cells overexpressing AccDpp and with the pET30-a(+) vector were grown in LB-kanamycin agar plates and incubated at 37°C for 45 min. Next, the agar plates were covered with five filter discs (6 mm in diameter), which were soaked with 2 μL of various concentrations of reagents. The reagents contained HgCl_2_ (0 mg/mL, 5 mg/mL, 10 mg/mL, 20 mg/mL, and 70 mg/mL), paraquat (0 mM, 50 mM, 200 mM, 300 mM, and 500 mM), CdCl_2_ (0 mM, 300 mM, 500 mM, 700 mM, and 900 mM), and cumene hydroperoxide (0 mM, 12.5 mM, 25 mM, 50 mM, and 100 mM). HgCl_2_ and CdCl_2_ were dissolved by water. Paraquat and cumene hydroperoxide were diluted by water and absolute ethyl alcohol, respectively. The treated cells were cultured overnight at 37°C.

### GenBank accession properties of the genes used in this paper

Many genes were used to perform bioinformatics analysis. Their species name and GeneBank accession number are listed in S4 Table.

## Results

### Characterization of *AccDpp*

**Fig 1:**
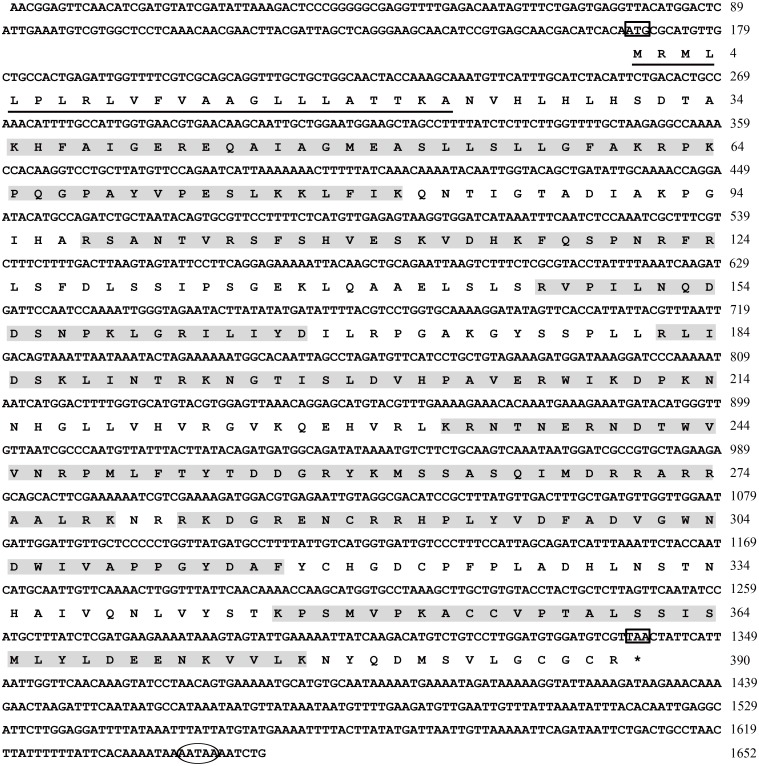
The cDNA sequence of *AccDpp* and its amino acid sequence. The top line shows the nucleotide sequence of *AccDpp*, and the second line shows the deduced amino acid sequence. The start codon (ATG) and stop codon (TAG) are boxed. The polyadenylation signal (AATAA) sequence is marked by an oval. The underlined region indicates the signal peptide, and the shaded amino acid sequence denotes the predicted antimicrobial peptide. The sequence was deposited in GenBank, and the GenBank accession no. is KT750952.

The full-length cDNA of *AccDpp* (GenBank accession number: KT750952) is 1,652 bp, with a 1,104-bp open reading frame (ORF) that encodes 390 amino acids. The amino acid sequence of AccDpp contains a signal peptide with 23 amino acids (Fig 1). Thus, the mature protein of *AccDpp* only contains 367 amino acids, and is a secretory protein. The molecular weight and theoretical pI of mature AccDpp was 41.38 kDa and 9.65, respectively. The *AccDpp* gene is flanked by a 167-bp 5ʾ untranslated region (5ʾ UTR) and a 312-bp 3ʾ UTR (Fig 1). In the 3ʾ UTR of *AccDpp*, a typical polyadenylation signal sequence (AATAA) existed.

**Fig 2:**
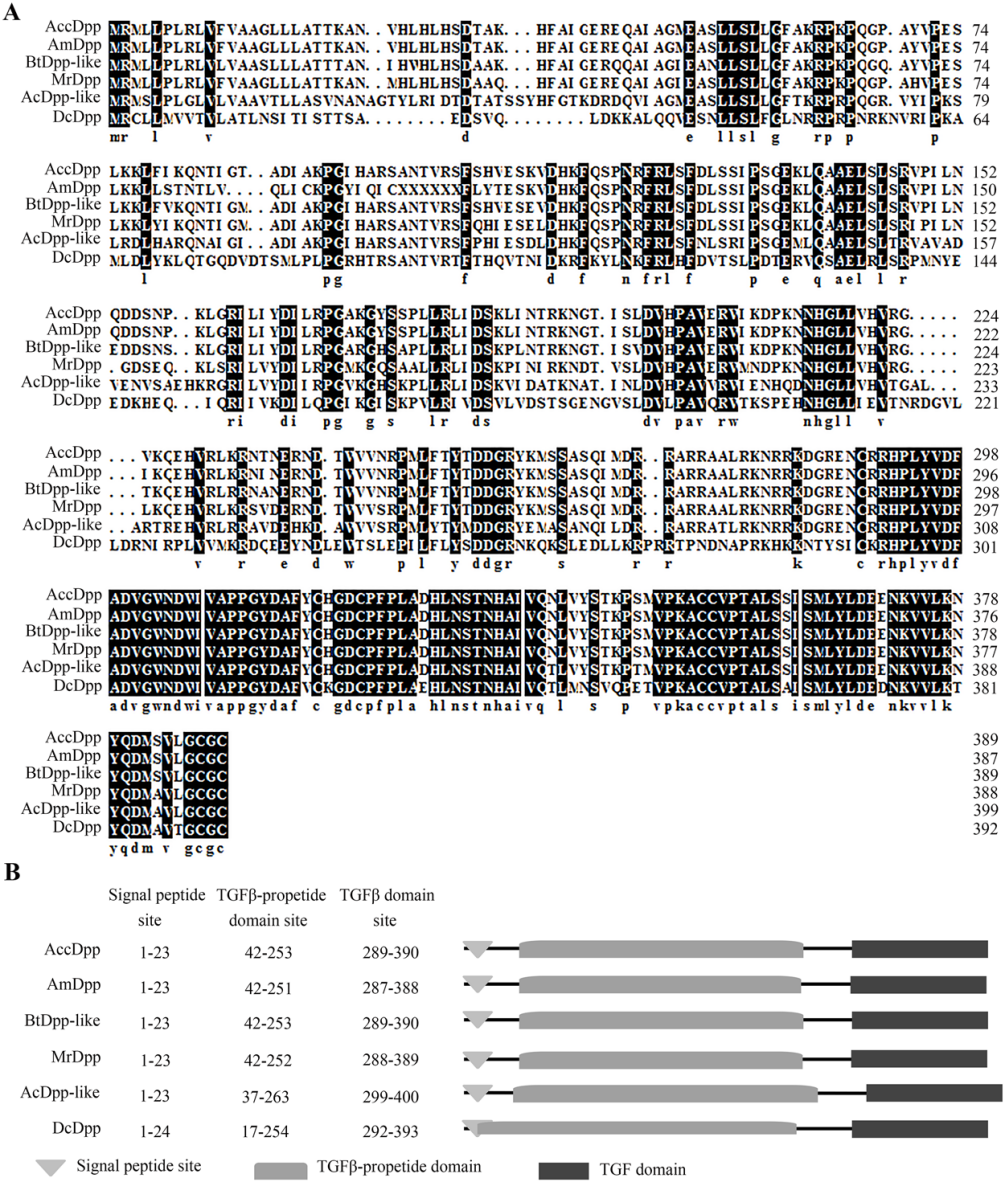
Characterization of *Dpp* from various species. The amino acid sequences of Dpp were all downloaded from the NCBI database (S4 Table). A, alignment of the deduced *AccDpp* protein sequence with other Dpp proteins. B, Conserved domain of Dpp. The conserved domains are marked by different shapes.

Fig 2A revealed that the C-terminus of Dpp of different species was highly conserved, while the N-terminus was not. The TGFβ-propeptide domain and TGFβ domain of Dpp in various species were predicted by the NCBI Conserved Domain Database. The results showed that the TGFβ-propeptide domain and TGFβ domain existed in the N-terminus and C-terminus of the Dpp protein, respectively (Fig 2B). The TGFβ-propeptide is known as a latency-associated peptide (LAP) in TGFβ. LAP is a homodimer that is disulphide linked to the TGFβ binding protein. The TGFβ domain is a multifunctional peptide that controls proliferation, differentiation, and other functions in many cell types. These two conserved domains may decide the functions of AccDPP in *A*. *cerana cerana*.

**Fig 3:**
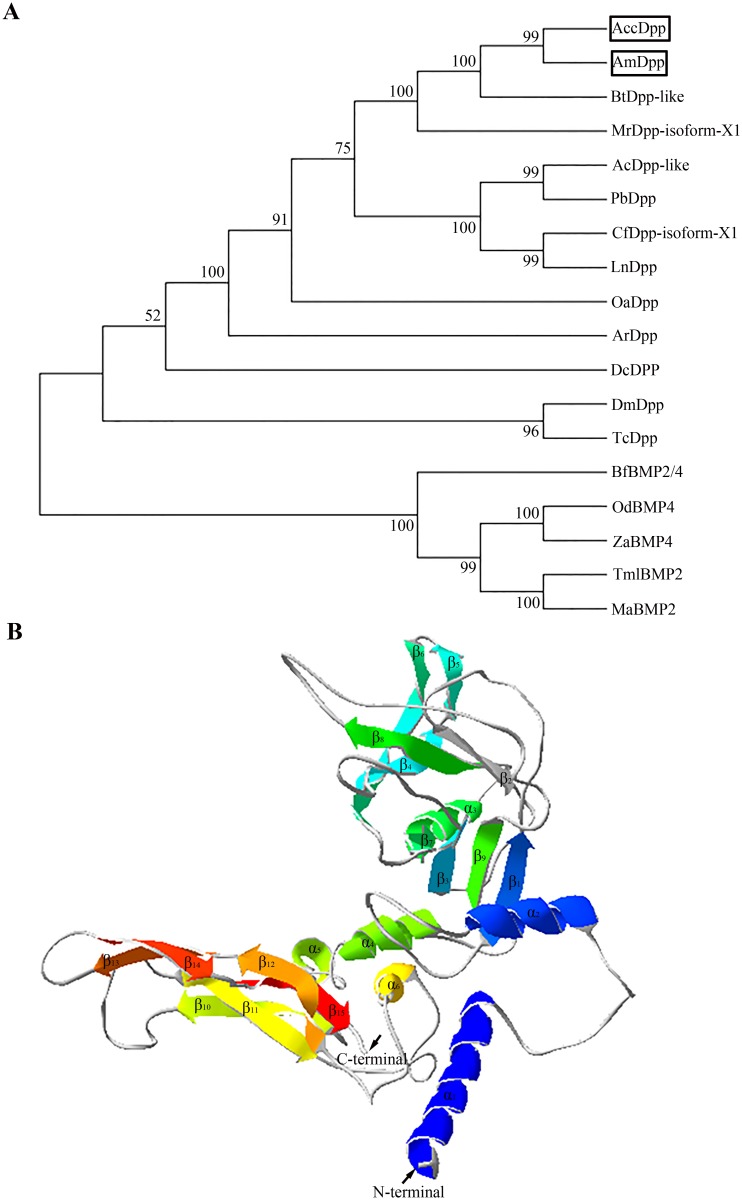
Phylogenetic analysis and the tertiary structure of *AccDpp*. A, phylogenetic analysis of AccDpp from different species. The species source of the above analysis is listed in S4 Table. B, the tertiary structure of AccDpp. Helices, sheets, and coils are presented in different colours.

A neighbour-joining phylogenetic tree was generated by MEGA 4.1 to explore the evolutionary relationships of Dpp among different species, and the result revealed that AccDpp was more closely related to AmDpp than other species (Fig 3A). Fig 3B showd the possible three-dimensional structure of AccDpp that may contribute to better understanding of the role of AccDpp. The subunit of AccDPP had more β folds (15) than α helices (6) (Fig 3B and S5 Table), that may be related to the function of AccDpp.

### The analysis of genetic sequence structure of *AccDpp*

**Fig 4:**
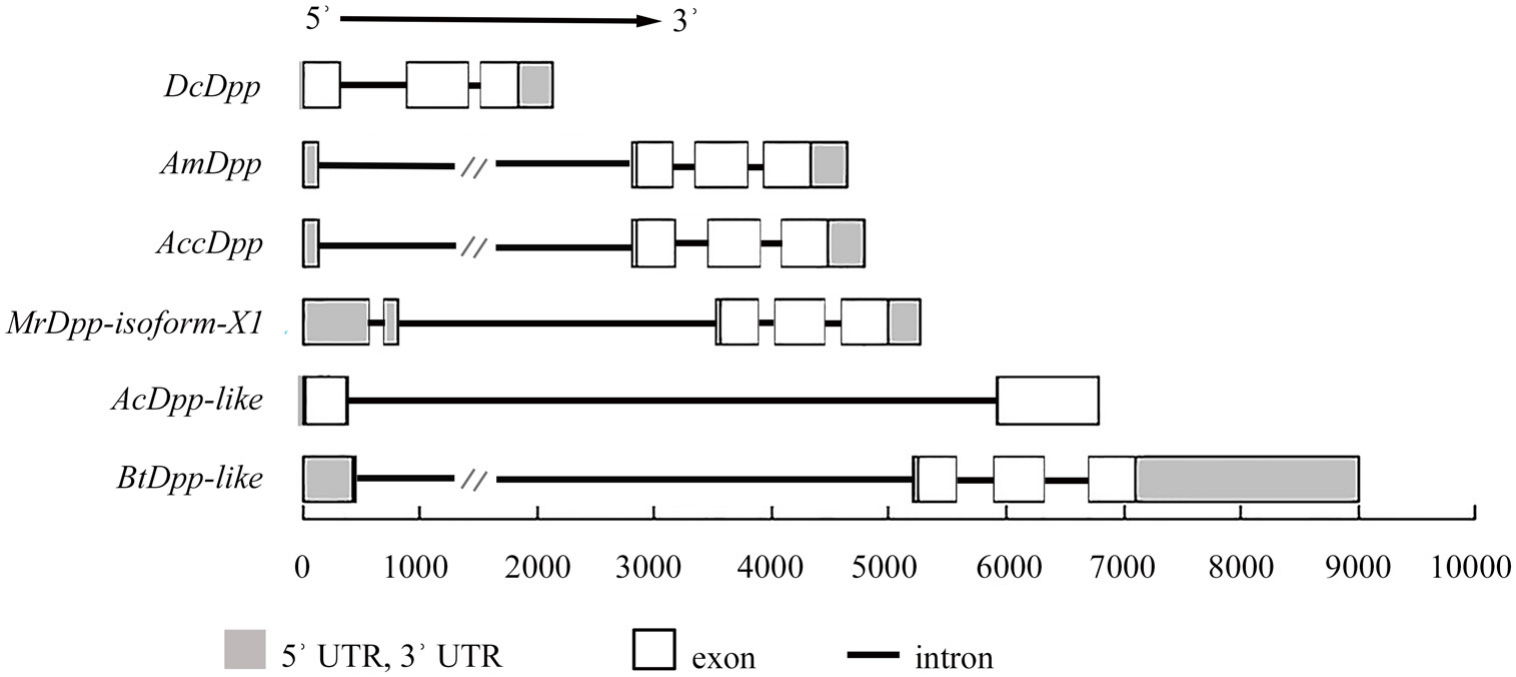
Schematic representation of the DNA structures of *Dpps*. The legend presents the pattern of untranslated regions, introns, and exons. Some introns of *Dpp* have been partially abridged in this figure for their partial sequence. The length of the genomic DNA of *Dpps* is loaded from the NCBI database, and their GeneBank accession numbers are listed in S4 Table.

A 4,066-bp sequence of *AccDpp* was isolated to study its genomic feature and included three introns and three exons (GenBank accession number: KT750953). It is interesting, a long intron was located inside the 5ʾ UTR of *AccDpp*. Both in this long intron and in the 5ʾ UTR contained many putative transcription factor binding sites (TFBs) (S1 Fig), including fifty-three CdxA, fourteen CF2-II, ten HSF, one NIT2, and one BR-C. Thus, this intron and 5ʾ UTR might be involved in the regulation of transcription of *AccDpp*. The GC content of the exons of *AccDpp* was higher than that of its intron (S6 Table), its similar with other *Dpp* genes. The size and GC content of Dpp exons from different species had a higher homology than its introns (Fig 4 and S6 Table), representing the conservation and variability of the same gene during evolutionary periods.

### Putative transcription factor binding sites on the *AccDpp* promoter

**Fig 5:**
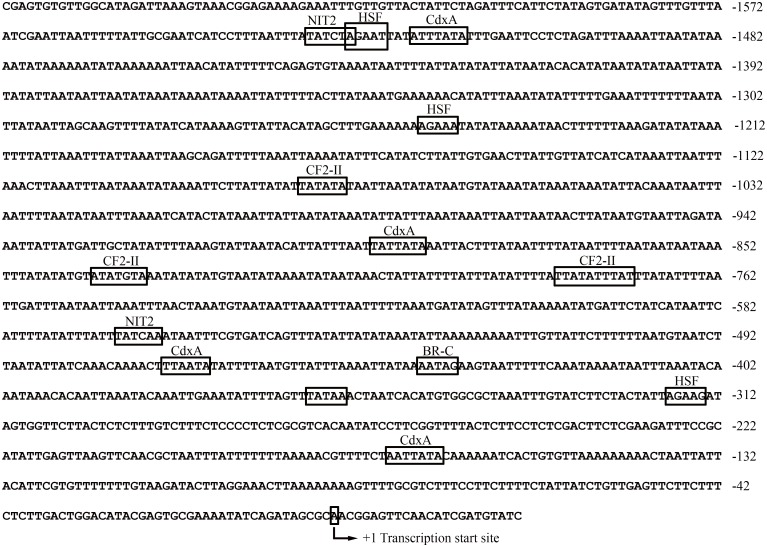
Partial nucleotide sequences and prediction transcription factor binding sites in the promoter region of *AccDpp*. The transcription start site and putative transcription factor binding sites mentioned in this paper are marked with arrows and boxes, respectively. The sequence was deposited in GenBank, and the GenBank accession no. is KT750953.

A 1,571-bp promoter sequence (GenBank accession number: KT750953) was isolated to investigate the organization of regulatory regions of *AccDpp*. As shown in Fig 5, fifty-three CdxA, seven CF2-II, six HSF, three NIT2, and one BR-C were identified in the promoter of *AccDpp*. Heat shock transcription factor (HSF) can respond to heat shock and contributes to building a cytoprotective state of the cell [44]. CdxA, CF2-II, NIT2, and BR-C are associated with embryo or tissue development [45, 46, 47, 48]. The results indicated that *AccDpp* might participate in organismal growth and various environmental stress responses.

### Expression and characterization of recombinant *AccDpp*

**Fig 6:**
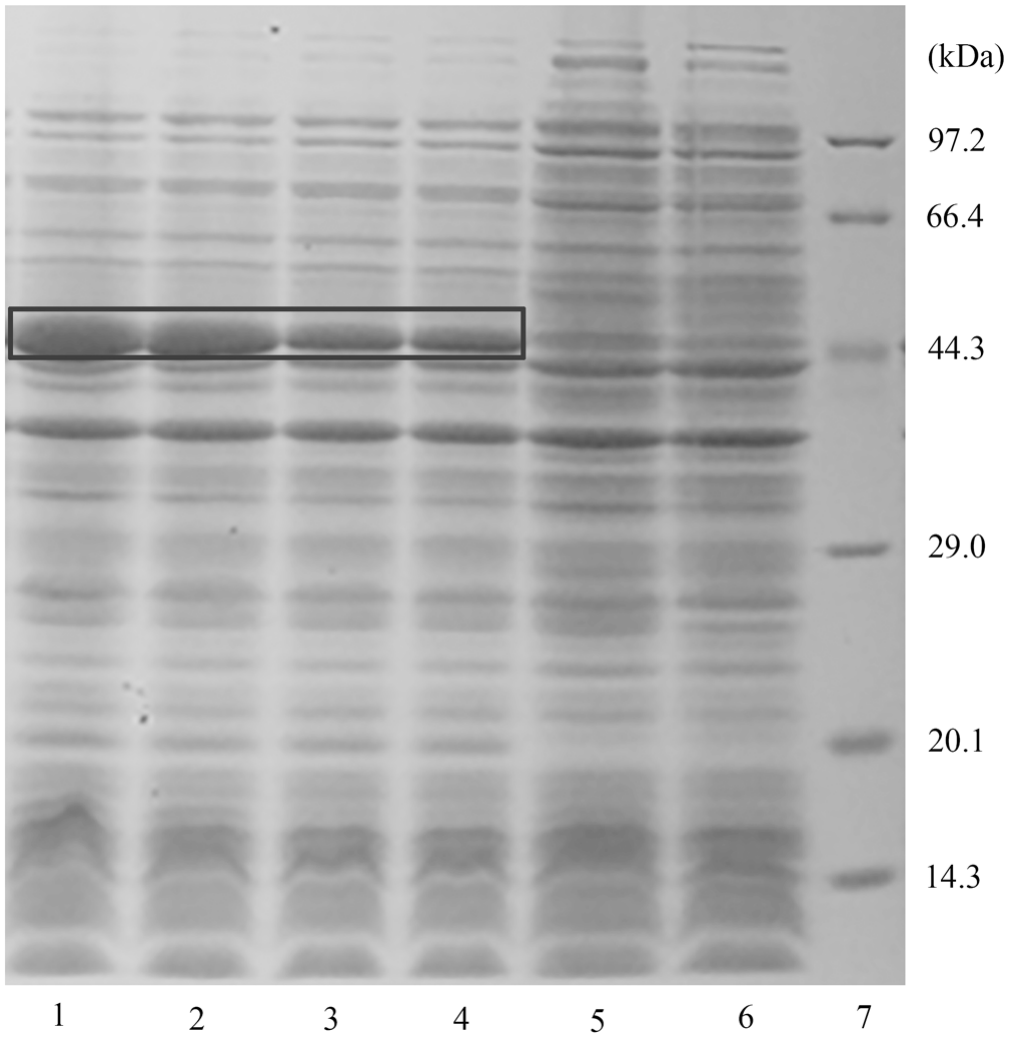
Expression of *AccDpp* in *Transetta* (DE3) chemically competent cells. Recombinant AccDpp was separated by SDS-PAGE, and then stained with Coomassie brilliant blue. Lane 1–4, expression of AccDpp after IPTG induction for 4, 5, 6, and 7 h. Lane 5 and Lane 6, non-induced of recombinant AccDpp and induced overexpression of pET-30a (+) vector for 7 h. The box shows the site of recombinant AccDpp.

*AccDpp* was overexpressed in *Transetta* (DE3) with two histidine tags and separated by SDS-PAGE. The recombinant protein had a molecular mass of 47.98 kDa, approximately 41.38 kDa attributed to AccDpp and approximately 6.60 kDa to cleavable N- and C-terminal His-tags (Fig 6). It is interesting that only when the signal peptide of AccDPP was removed could recombinant AccDpp be induced by IPTG. The signal peptide can guide the nascent polypeptide chain across the endoplasmic reticulum; however, *E*. *coli* does not have an endoplasmic reticulum. Moreover, the recombinant AccDpp was almost insoluble in *Transetta* cells, and the HisTrap^™^ FF column could not purify the recombinant AccDpp (data not shown).

### Temporal and spatial expression of *AccDpp* and its protein

**Fig 7:**
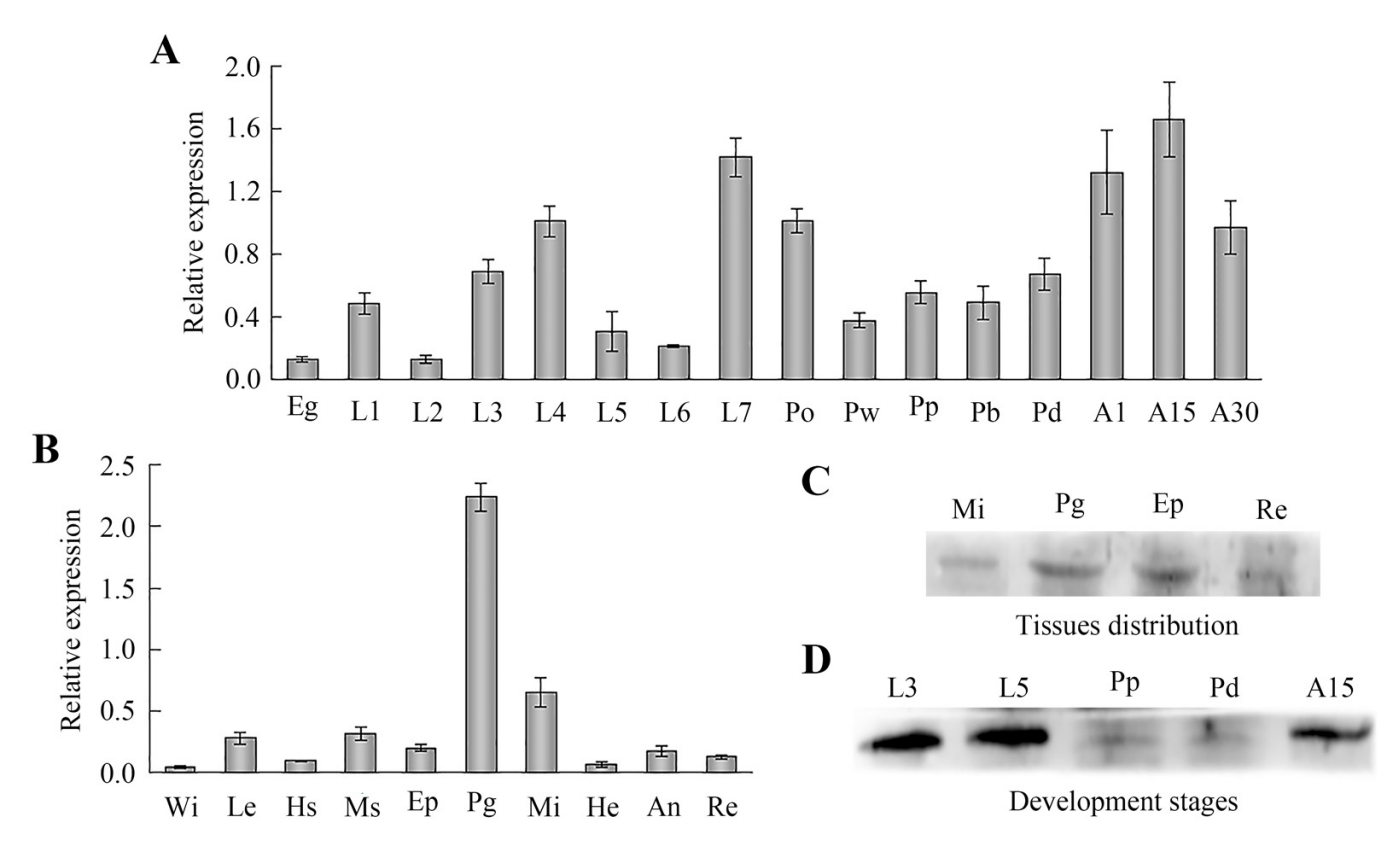
Expression profile of *AccDpp* and Western blot analysis of *AccDpp* at different developmental stages and different tissues. A, and B, the mRNA level of *AccDpp* at different developmental stages: egg (E), one-day to seven-day larvae (L1-L7), pre-pupal phase pupae (Po), pupae (white-eyed (Pw), pink-eyed (Pp), brown-eyed (Pb) and dark-eyed (Pd) pupae)), 1-day worker bees (A1), 15-day worker bees (A15), and 30-day worker bees (A30), and different tissues: leg (Le), wing (Wi), muscle (Ms), midgut (Mi), haemolymph (He), rectum (Re), poison gland (Pg), honey sac (Hs), antennae (An) and epidermis (Ep), respectively. The data are the mean ± SE of three independent experiments. The letters above the columns suggest significant differences (P<0.0001) according to Duncan’s multiple range tests. C, and D, the expression level of AccDpp protein at different tissues and stages of development, separately.

Various developmental stages and tissue expression profiles of *AccDpp* were investigated by qRT-PCR. *AccDpp* had expression at all stages and was highly expressed in 15-day adult bees (Fig 7A). *AccDpp* was expressed in all of the selected tissues, while highest expression level in the poison gland, followed by the midgut (Fig 7B). Western blotting was performed to explore the AccDpp content in different tissues. As shown in Fig 7C, the expression of AccDpp was higher in the poison gland than in the epidermis, rectum, and midgut. Western blotting was also used to detect the protein level of AccDpp at stages L3, L5, Pp, Pb, and A15. The results showed that the content of AccDpp protein was higher in stage L3 and L5, followed by stage A15, Pp, and Pb (Fig 7D), a result that was not completely consistent with the qRT-PCR findings. These data revealed that *AccDpp* might be related to development and growth in *A*. *cerana cerana*.

### Expression patterns of *AccDpp* under different types of abiotic stresses

**Fig 8:**
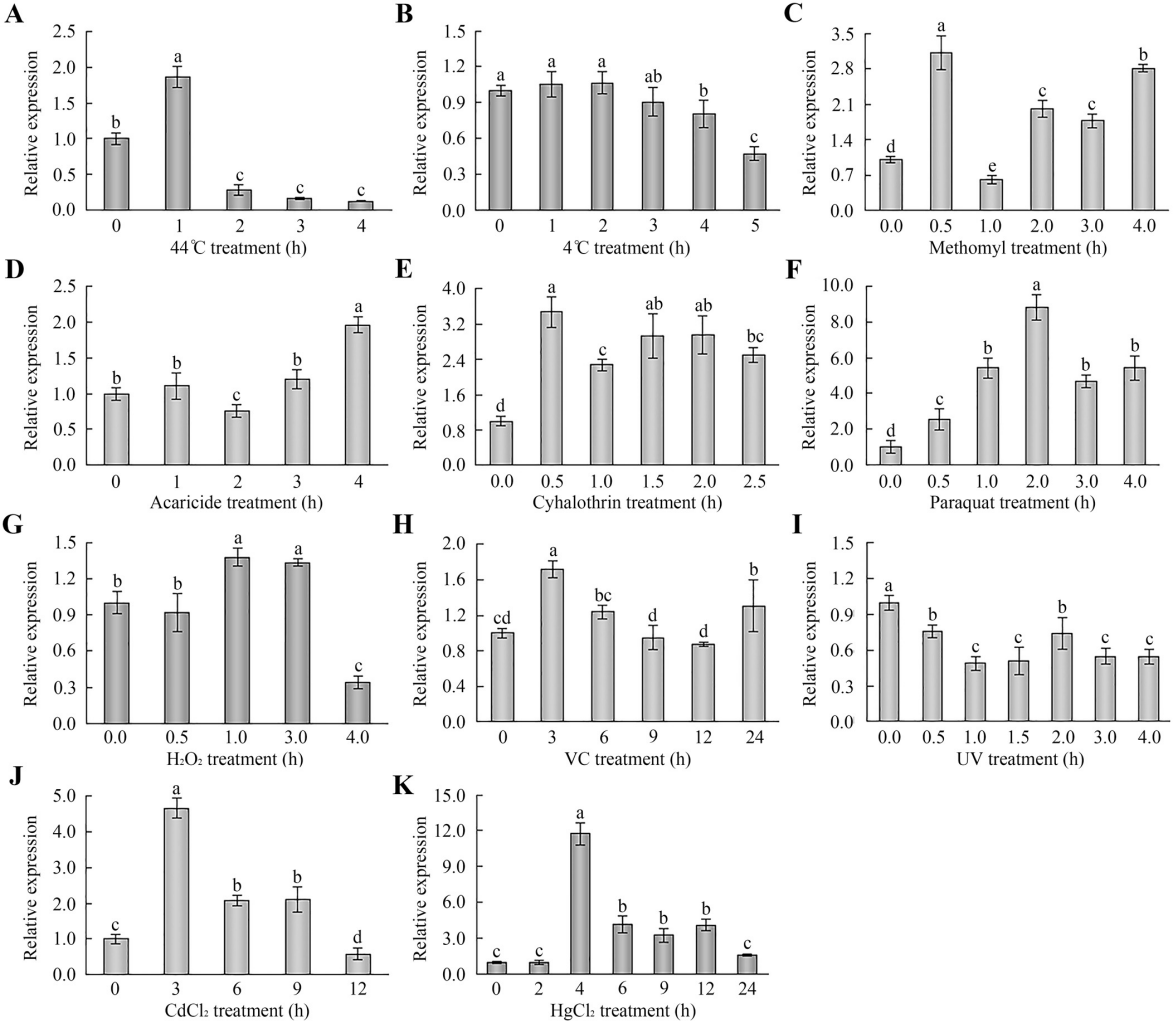
Expression of *AccDpp* under different stress conditions. The transcript levels of *AccDpp* were analysed via qRT-PCR. Untreated 15-day worker bees and the *β-actin* gene were used as controls and an internal control, separately. The data are the mean ± SE of three independent experiments. Significant differences (p<0.001) were represented by different letters on the bar based on Duncanʾs multiple range tests.

Although the expression level of AccDpp protein was higher in the L3 and L5 stages than in the A15 stage (Fig 7D), the larvae were not easily bred, and its quantity was less. Moreover, the mRNA level of the A15 stage was higher than of the other stages (Fig 7A). Therefore, the 15-day worker bees were selected to be treated with 4°C, 44°C, H_2_O_2_, UV, VC, acaricide, cyhalothrin, paraquat, methomyl, HgCl_2_, and CdCl_2_. As shown in Fig 8A and 8B, the mRNA level of *AccDpp* was induced and repressed after 44°C and 4°C treatment, respectively, and reached maximums and minimums at 1 h and 5 h, separately. When the 15-day worker bees were exposed to methomyl, acaricide, cyhalothrin, and paraquat, the transcript levels of *AccDpp* were all upregulated and accumulated to their highest level at 0.5 h, 4 h, 0.5 h, and 2 h (Fig 8C–8F). Under stress using with H_2_O_2_ and VC, the mRNA expression of *AccDpp* was slightly increased at 1 h and 3 h (Fig 8G and 8H), respectively, compared with the control. Conversely, the expression of *AccDpp* was reduced after UV treatment (Fig 8I). When the 15-day worker bees were fed with food containing CdCl_2_ and HgCl_2_, the transcript levels of *AccDpp* were increased 4.67-fold and 11.74-fold compared to untreated honey bees, respectively, although the mRNA levels of *AccDpp* were gradually down-regulated over time (Fig 8J and 8K). The above results indicated that *AccDpp* might participate in a stress response.

### Western blot analysis of *AccDpp* under abiotic stress conditions

**Fig 9:**
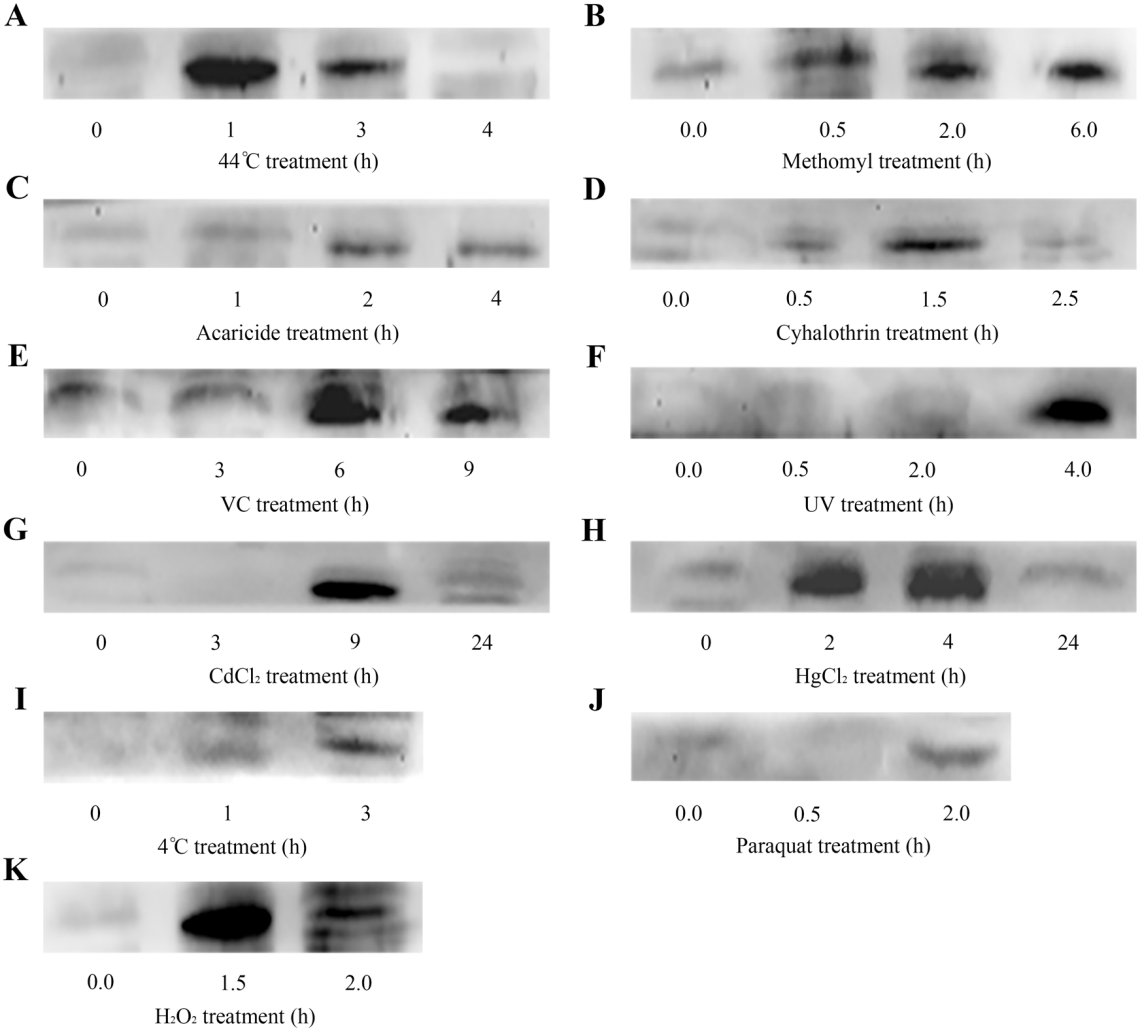
Western blot analysis of *AccDpp*. Fifteen-day-old adult bees were exposed to 44°C (A), methomyl (B), acaricide (C), cyhalothrin (D), VC (E), UV (F), CdCl_2_ (G), HgCl_2_ (H), 4°C (I), Paraquat (J), and H_2_O_2_ (K). An equivalent concentration of extracted protein was loaded in every lane under the same treatment conditions.

Further studies (Western blot analysis) aimed at exploring the level of AccDpp under the condition of abiotic stress. On the whole, the amount of AccDpp was increased to a certain degree after exposure to all of the stressful agents (Fig 9), although the time and extent of induction period showed some differences with the expression patterns of *AccDpp* under the same stress conditions. After being subjected to 44°C for 1, 3, and 4 h, the level of AccDpp reached a peak at 1 h (Fig 9A), which consistent with the result of qRT-PCR. Following exposure to methomyl, acaricide, cyhalothrin, and VC, the expression level of AccDpp increased at different times (Fig 9B, 9C, 9D and 9E). UV, CdCl_2_ and HgCl_2_ potently enhanced the amount of AccDpp protein at 4.0 h, 9 h, and 4 h (Fig 9F, 9G and 9H), respectively. As shown in Fig 9I, in contrast to the mRNA level of *AccDpp*, AccDpp accumulated at 3 h when 15-day bees were subjected to 4°C. Paraquat treatment did not caused noticeable increases in the protein level of AccDpp (Fig 9J). The expression level of AccDpp was potently enhanced at 1.5 h (Fig 9K) under the treatment of H_2_O_2_. These findings revealed that AccDpp might play a pivotal role when *A*. *cerana cerana* was subjected to stress stimuli.

### Disc fusion assay of recombinant *AccDpp*

**Fig 10:**
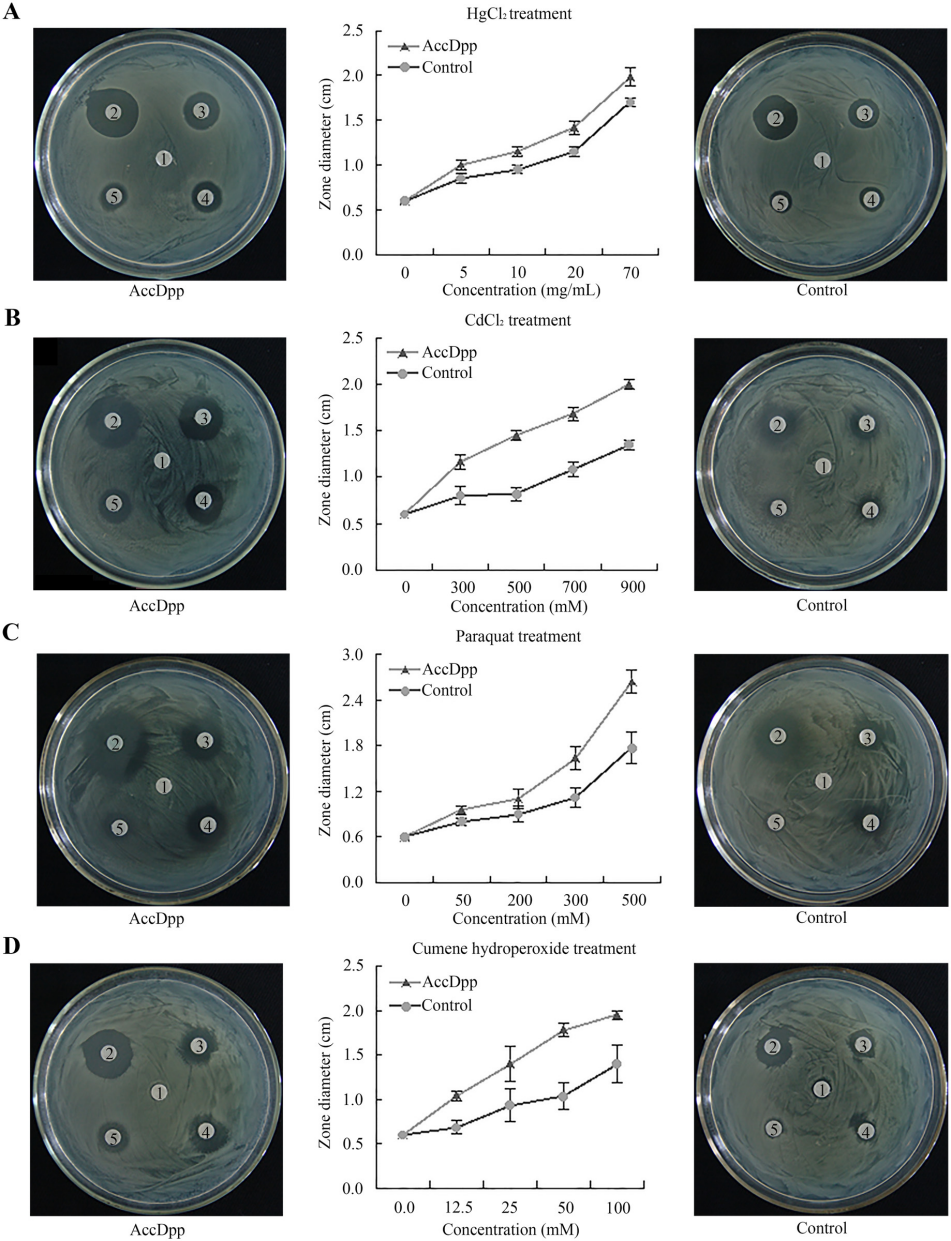
Disc diffusion assays of overexpressed recombinant *AccDpp*. The selected reagents are HgCl_2_, CdCl_2_, paraquat, and cumene hydroperoxide. The numbers on the filter discs from 2–5 represent the concentration of reagents from small to large, and the number 1 indicates the control. The data are the mean ±SE of three independent experiments.

Recombinant AccDpp protein was exposed to four reagents to provide further evidence that AccDpp was related to the stress response. *E*. *coli* with pET-30a (+) vector used as the control. The results showed that the killing zones were larger around the filters on the plates with cells overexpressing AccDpp than around the filters of the control plates (Fig 10), a finding that was opposite to the expected results. This suggested that AccDpp might have antimicrobial activity. The antimicrobial peptide database was used to predict the antimicrobial activity of the peptide in AccDpp. Most of active antimicrobial peptides have a net charge between +3 to +8. However, peptide that having a neutral charge may also have antimicrobial activity. The predictable results showed that there were at least seven antimicrobial peptides in the sequence of AccDpp (Fig 1). These data indicated that AccDpp was likely to have antibacterial activity.

## Discussion

The TGFβ superfamily is associated not only with the growth, differentiation, and apoptosis of cells but also with ROS. *Dpp* belongs to the TGFβ superfamily. Studies in model organisms have suggested that *Dpp* notably contributes to the body axis decision and the development of appendages [49, 50]. However, few reports have discussed the role of *Dpp* in the ROS response in insects.

In this paper, we used the Chinese bee as an experimental insect and successfully isolated *Dpp* gene (*AccDpp*). The ORF of *AccDpp* encoded 390 amino acids, which included a signal peptide consisting of 23 amino acids (Fig 1). The sequence of the C-terminus of Dpp from different species was highly conserved compared with that of the N-terminus (Fig 2A). The TGFβ domain of Dpp from different species consisted of 101 amino acids (Fig 2A and 2B). The results of Fig 2A and 2B suggested that the TGFβ domain and TGFβ-propeptide domain might decide the conservation and diversity of function of Dpp protein among various species during the course of evolution, respectively. Phylogenetic analysis showed that AccDpp presented the closest evolutionary relationships with AmDpp (Fig 3A). The sequence identity of AccDpp and AmDpp can reach 92.82%. Such a high sequence identity is also present in other genes of *A*. *cerana cerana* [51, 52, 53, 54, 55] and *A*. *mellifera*, and some protein sequences are even exactly the same. However, the traits and characteristics of these two bees are very different, possibly due to the environment and minor differences between the genes.

Additionally, a 1,571-bp promoter sequence of *AccDpp* was cloned. Sequence analysis showed that there are many transcription factor binding sites (TFBs) in the promoter (Fig 5) that play a role in development and the stress response. It is worth mentioning that a intron more than 2,000-bp sequence presented inside the 5΄ UTR of *AccDpp*. Many TFBs existed in this intron and the 5΄ UTR of *AccDpp* (S1 Fig), which may also control the transcription of *AccDpp* as its promoter. Such a long intron also exists in the coding region of *Dpp* of other species (Fig 4 and S6 Table). Recent evidence had shown that the expression of *Dpp* was extremely complicated and could be regulated by the adjustment of the 5΄ and 3΄ coding region, and a 50-kb intron (this intron interrupted the protein coding region) in *D*. *melanogaster* [56, 57]. This long intron can also exist inside the 5΄ UTR of *Dpp* in other species. The different positions of it may be the result of the evolution of species. Research had demonstrated that *Dpp* played a role in developmental processes [33] and was expressed at all of the development stages of *Polyrhachis vicina Roge*, *D*. *melanogaster*, and *Bombyx mori*. qRT-PCR analysis showed that *AccDpp* was expressed from the egg to adult and had the highest transcript level at the A15 stage in *A*. *cerana cerana*, suggesting that *AccDpp* participated in Chinese bee development (Fig 7A), which was not consistent with the Western blotting result (Fig 7D). The same result was obtained in the expression pattern of *AccDpp* in different tissues (Fig 7B and 7C). That may be due to the particular body needs of *AccDpp* at the mRNA and protein levels. The poison gland, midgut, and epidermis are associated with self-defence, protection from oxidative damage and exogenous substance detoxification [58], and the stabilization of physical as well as stress response [59], respectively. The tissue-specific expression of *AccDpp* indicated it may have protective activity against the impairment of environmental stress and xenobiotics.

The above results prompted us to explore the role of *AccDpp* under oxidative stress conditions. Abrashev et al. (2008) suggested that heat shock could induce the antioxidant response and oxidative stress [60]. A decrease in the temperature leads to the transcription and translation of many genes, including genes that are induced following ROS induction. The antioxidative and metabolic systems were changed after exposure to cold stress in rats [61]. ROS can also be induced by the accumulation of toxic pesticides, resulting in oxidative injury in the living body [62, 63]. For example, the early embryonic development of amphibians was seriously affected by the widespread use of paraquat, which could induce ROS generation. The processes related to cell aging were intensely affected by adding pesticides in the culture of yeast *Saccharomyces cerevisiae* [64], likely because pesticides induce oxidative lesions by stimulating the production of free radicals. UV irradiation provokes ROS formation, leading to the activation of complex signalling pathways, such as mitogen activated protein kinase (MAPK) and nuclear factor kappa-β (NF-кβ) pathways, finally causing cellular death [4, 65]. H_2_O_2_ is one of the three major types of ROS, resulting directly from the action of oxidase enzymes or from the dismutation of superoxide anion radicals [66]. Experimental evidence had indicated that DNA oxidative lesions and mutation can be induced by cadmium, which could influence cell proliferation, differentiation, and apoptosis and might be associated with carcinogenesis [6, 67]. Several studies had indicated that mercury played a role in the generation of oxygen radicals [68, 69]. As an antioxidant, vitamin C (VC) can mitigate oxidative stress [70]; however, VC can also cause oxidative damage of DNA [71]. Thus, we can see that heat, cold, pesticide, heavy metals, UV, H_2_O_2_, and VC are all related to oxidative stress. So we selected 4°C, 44°C, acaricide, cyhalothrin, paraquat, methomyl, HgCl_2_, CdCl_2_, VC, UV and H_2_O_2_ to simulate oxidative stress conditions to treat *A*. *cerana cerana* and test the response of *AccDpp*.

*AccDpp* expression might be related with temperature (4°C) and UV stress (Fig 8B and 8I), but not enough to prevent the translation of *AccDpp* (Fig 9I and 9F). The transcript levels of *AccDpp* were elevated after exposure to 44°C (Fig 8A), methomyl (Fig 8C), acaricide (Fig 8D), cyhalothrin (Fig 8E), H_2_O_2_ (Fig 8G), and CdCl_2_ (Fig 8J) to a certain degree, although its comparative expression profile varied in response to different conditions, suggesting that *AccDpp* might be relevant to the oxidative stress response. VC treatment increased the mRNA level of *AccDpp* (Fig 8H). We speculated that the dose of VC was sufficient to induce *AccDpp* to participate in the reaction of ROS. Moreover, the transcript levels of *AccDpp* were significantly enhanced by paraquat and HgCl_2_ (Fig 8F and 8K), indicating that paraquat and HgCl_2_ were more conducive to the translation of *AccDpp*. Our transcriptional analysis of *AccDpp* suggested that *AccDpp* might play a role in oxidative stress.

Furthermore, Western blotting was performed to explore the protein level of *AccDpp* when *A*. *cerana cerana* was subjected to other oxidative pressures, including 44°C, methomyl, acaricide, cyhalothrin, VC, UV, CdCl_2_, HgCl_2_, and H_2_O_2_. The findings indicated that AccDpp expression was enhanced under these conditions compared with the untreated group. The extent of induction of AccDpp was more obvious under 44°C, H_2_O_2_, VC, UV, HgCl_2_, and CdCl_2_ conditions. It is noteworthy that the induced degree and time point of *AccDpp* showed a sensible difference at the mRNA and protein levels. Although mRNA and its corresponding protein both exist in the cell, only the protein plays a role. ROS could increase TGFβ expression, and TGFβ could mediate the production of ROS [16, 17, 18, 20]. Thus, we suggest that AccDpp is implicated in the oxidative stress response.

Concerning the protein levels of *AccDpp* that were not consistent with its transcriptional patterns, the following explanations should be considered. First, the increased level of AccDpp could be a result of the accumulation of protein. Although the transcription of *AccDpp* was repressed, the already existing mRNA could continue to be translated. Second, the simulated environmental stress regulated the transcription and translation of *AccDpp* through different signal transduction pathways. Third, that is a result of posttranscriptional regulation. A recent paper reported that, although the *invE* mRNA was readily detectable, the expression of its protein was tightly repressed. Mitobe et al. (2009) reported that RNA-binding protein Hfq was involved in the regulation of *invE* gene expression through posttranscriptional regulation [72]. Last but not least, there are several RNAs involved in mRNA transcription and translation, such as miRNAs and circRNAs. For example, miRNAs are implicated in the regulation of many pivotal processes of enamel maturation by affecting mRNA translation and stability in rat incisors [73]. Many studies had demonstrated that circRNAs could regulate the splicing, transcription, posttranscription, and activation of protein [74, 75]. The difference in the expression profiles of *AccDpp* and AccDpp may due to their regulation by miRNAs and circRNAs.

To evaluate whether recombinant AccDpp has an antioxidant function in *E*. *coli* cells, disc diffusion assays were performed. However, the findings showed that the killing zones were not smaller around the filters on the plates with cells overexpressing recombinant AccDpp than around the filters on the control plates (Fig 10). A previous study reported that recombinant arginine kinases of *A*. *cerana cerana* could inhibit the growth of bacteria, which because of the antimicrobial peptide in arginine kinase protein [37]. The antimicrobial peptides not only had broad-spectrum anti-bacterial activity but also high antibacterial activity [76]. Therefore, we considered the antibacterial activity of the antimicrobial peptides of AccDpp led to the result of the disc diffusion assay experiment. The antimicrobial peptides play an important role in the humoral immune defence [77]. The TGFβ superfamily can participate in the immune response of organisms [14, 15]. The antimicrobial peptides of AccDpp may cause AccDpp to participate in the immune response.

Collectively, these results provided evidence that *AccDpp* might play a role in the development of *A*. *cerana cerana* and oxidative stress response. Findings of this present reported will be conducive to studying the development of Chinese bees and other insects. This will be provide a foundational knowledge to explore and understand the TGFβ signal transduction pathway in the future.

## Supporting Information

S1 Fig The partial sequence of a long intron and 5ʾ UTR of *AccDpp* and the predicted transcription factor binding sites in its region. The transcription start site and translation start site are marked with arrows. The putative transcription factor binding sites implicated in this research are denoted with boxes. The 5ʾ UTR region is signified by the shaded area. The sequence was deposited in GenBank, and the GenBank accession no. is KT750953.
(TIF)

S1 Table The abiotic stress condition to *A*. *cerana cerana*. (DOC)

S2 Table Primer sequences used in this research. (DOC)

S3 Table Procedures used in this study. (DOC)

S4 Table Characterization of gene used in this paper. (DOC)

S5 Table The basic subunit of *AccDpp* secondary structure. (DOC)

S6 Table Exons and introns size and GC content of the deosited *Dpp* gene in NCBI. (DOC)

## Abbreviations

TGFβ: Transforming growth factor β
BMP: Bone morphogenetic protein
GDF: Growth differentiation factor
Dpp: Decapentapletic gene
A. cerana cerana: Apis cerana cerana
A. Mellifera: Apis mellifera
D. Melanogaster: Drosophila melanogaster
Bp: Base pair
E. coli: *Escherichia coli*
IPTG: Isopropyl-P-D-thiogalactoside
LB: Luria-bertani medium
qRT-PCR: Quantitative real time PCR
RACE: Rapid amplification of cDNA ends
ROS: Reactive oxygen species
SDS: Sodium dodecyl sulfate
SDS-PAGE: SDS Poly-acrylamide gel electrophoresis
VC: Vitamin C
