# Supplementary figures and images for: Characterization of a Decapentapletic Gene (AccDpp) from Apis cerana cerana and Its Possible Involvement in Development and Response to Oxidative Stress
Source: PLoS One. 2016 Feb 16;11(2):e0149117. doi: 10.1371/journal.pone.0149117 (PMC4755538; doi:10.1371/journal.pone.0149117)

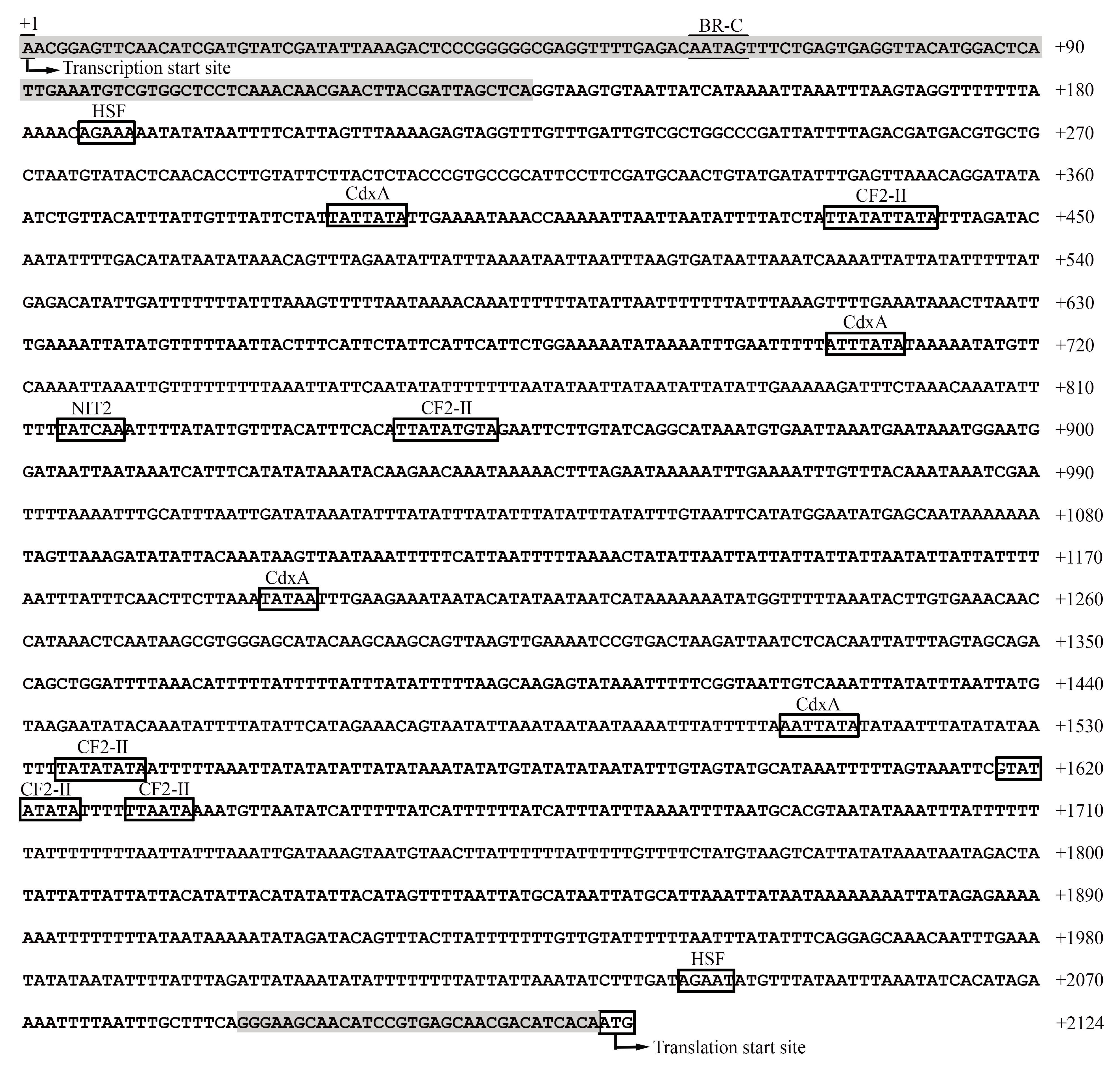

Supplement: S1 Fig — The transcription start site and translation start site are marked with arrows. The putative transcription factor binding sites implicated in this research are denoted with boxes. The 5ʾ UTR region is signified by the shaded area. The sequence was deposited in GenBank, and the GenBank accession no. is KT750953. (TIF) [file pone.0149117.s001.tif]
