# Supplementary material for: Characterization of a Decapentapletic Gene (AccDpp) from Apis cerana cerana and Its Possible Involvement in Development and Response to Oxidative Stress
Source: PLoS One. 2016 Feb 16;11(2):e0149117. doi: 10.1371/journal.pone.0149117 (PMC4755538; doi:10.1371/journal.pone.0149117)
Supplement: S1 Table — (DOC) [file pone.0149117.s002.doc]

S1 Table. The abiotic stress condition to *Apis cerana cerana*.

| Experiment condition | Treatment method | Collection time after treatment |
| --- | --- | --- |
| 44 °C | Exposure | 0, 1, 2, 3, and 4 h |
| 4 °C | Exposure | 0, 1, 2, 3, 4, and 5 h |
| Methomyl (1.8 g/L) | Exposure | 0.0, 0.5, 1.0, 2.0, 3.0, and 4.0 h |
| Acaricide (0.8 g/L) | Exposure | 0, 1, 2, 3, and 4 h |
| Cyhalothrin (25 mg/L) | Exposure | 0.0, 0.5, 1.0, 1.5, 2.0, and 2.5 h |
| Paraquat (25 mg/L) | Exposure | 0.0, 0.5, 1.0, 2.0, 3.0, and 4.0 h |
| H_2_O_2_ (2 mM, 0.5 uL/work) | injection | 0.0, 0.5, 1.0, 3.0, and 4.0 h |
| VC (0.02 g/ml) | feed | 0, 3, 6, 9, 12, and 24 h |
| UV (30 mJ/cm^2^) | Exposure | 0.0, 0.5,1.0, 1.5, 2.0, 3.0, and 4.0 h |
| CdCl_2_ (3 mg/mL) | feed | 0, 3, 6, 9, and 12 h |
| HgCl_2_ (3 mg/mL) | feed | 0, 2, 4, 6, 9, 12, and 24 h |
| Experiment condition | Treatment method | Collection time after treatment |
| 44 °C | Expourse | 0, 1, 2, 3, and 4h |
| 4 °C | Expourse | 0, 1, 2, 3, 4, and 5h |
| Methomyl (1.8g/L) | Expourse | 0.0, 0.5, 1.0, 2.0, 3.0, and 4.0h |
| Acaricide (0.8/L) | Expourse | 0, 1, 2, 3, and 4h |
| Cyhalothrin (25mg/L) | Expourse | 0.0, 0.5, 1.0, 1.5, 2.0, and 2.5h |
| Paraquat (25mg/L) | Expourse | 0.0, 0.5, 1.0, 2.0, 3.0, and 4.0h |
| H_2_O_2_ (2mM, 0.5uL/work) | injection | 0.0, 0.5, 1.0, 3.0, and 4.0h |
| VC (0.02 g/ml) | feed | 0, 3, 6, 9, 12, and 24h |
| UV (30mJ/cm^2^) | Expourse | 0.0, 0.5,1.0, 1.5, 2.0, 3.0, and 4.0h |
| CdCl_2_ (3 mg/mL) | feed | 0, 3, 6, 9, and 12h |
| HgCl_2_ (3 mg/mL) | feed | 0, 2, 4, 6, 9, 12, and 24h |
