# Supplementary material for: Characterization of a Decapentapletic Gene (AccDpp) from Apis cerana cerana and Its Possible Involvement in Development and Response to Oxidative Stress
Source: PLoS One. 2016 Feb 16;11(2):e0149117. doi: 10.1371/journal.pone.0149117 (PMC4755538; doi:10.1371/journal.pone.0149117)
Supplement: S2 Table — (DOC) [file pone.0149117.s003.doc]

**S2 Table.** Primer sequences used in this research.

| Abbreviation | Primer sequence (5′–3′) | Description |
| --- | --- | --- |
| ADF | GACATCACAATGCGCATGTTGC | cDNA sequence primer, forward |
| ADR | TTAACGACATCCACATCCAAGG | cDNA sequence primer, reverse |
| AD3RO | CCGCTTTATGTTGAC | 3′RACE forward primer, outer |
| AD3RI | GGTGATTGTCCCTTTCCATTAGCA | 3′RACE forward primer, inner |
| B26 | GACTCTAGACGACATCGA(T)18 | 3′RACE universal primer, outer |
| B25 | GACTCTAGACGACATCGA | 3′RACE universal primer, inner |
| AD5RO | CGAGAAAGACTTAATTCTGCAG | 5′RACE reverse primer, outer |
| AD5RI | CAACATGAGAAAAGGAACGCACTG | 5′RACE reverse primer, inner |
| AAP | GGCCACGCGTCGACTAGTAC(G)16 | Abridged Anchor Primer |
| AUAP | CATGGGTCAAACTTGTACAGATCG | Abridged universal amplification primer |
| ADPETF | GGTACCAATGTTCATTTGCATCTACATTCTG | Primers of constructing vector, forward |
| ADPETR | GAGCTCACGACATCCACATCCAAGGAC | Primers of constructing vector, reverse |
| ADPF | CGAGTGTGTTGGCATAGATTAAAGT | Promoter specific primer, forward |
| ADPR | GCGCTATCTGATATTTTCGCACTC | Promoter specific primer, reverse |
| ADRTF | ACAAGGTCCTGCTTATGTTCC | Real-time PCR primer, forward |
| ADRTR | CAGTGCGTTCCTTTTCTCATG | Real-time PCR primer, reverse |
| *β-s* | TTATATGCCAACACTGTCCTTT | Standard control primer, forward |
| *β-x* | AGAATTGATCCACCAATCCA | Standard control primer, reverse |
| G1 | ACAAGGTCCTGCTTATGTTCC | Genomic sequence primer, forward |
| G2 | TGCTAATGGAAAGGGACAATCACC | Genomic sequence primer, reverse |
| G3 | CGGAGTTCAACATCGATGTATCG | Genomic sequence primer, forward |
| G4 | CTGATACAAGAATTCTACATATAATGTGAAATG | Genomic sequence primer, reverse |
| G5 | CATTTCACATTATATGTAGAATTCTTGTATCAG | Genomic sequence primer, forward |
| G6 | CTAGCTGTCTGCTATTAAATAATTGTGAG | Genomic sequence primer, reverse |
| G7 | TACTTGTGAAACTGCCATAAACTC | Genomic sequence primer, forward |
| G8 | GGAACATAAGCAGGACCTTGTG | Genomic sequence primer, reverse |
