# Supplementary material for: Characterization of a Decapentapletic Gene (AccDpp) from Apis cerana cerana and Its Possible Involvement in Development and Response to Oxidative Stress
Source: PLoS One. 2016 Feb 16;11(2):e0149117. doi: 10.1371/journal.pone.0149117 (PMC4755538; doi:10.1371/journal.pone.0149117)
Supplement: S3 Table — (DOC) [file pone.0149117.s004.doc]

**S3 Table.** Procedures used in this study.

| Primers pair | Amplification conditions |
| --- | --- |
| ADF/ADR | 10 min at 94 °C, 40 s at 94 °C, 40 s at 53.2 °C, 1 min 20 s at 72 °C for 35 cycles, 10 min at 72 °C |
| AD3RO/B26 | 10 min at 94 °C, 40 s at 94 °C, 40 s at 53.2 °C, 1 min at 72 °C for 35 cycles, 10 min at 72 °C |
| AD3RI/B25 | 10 min at 94 °C, 40 s at 94 °C, 40 s at 55.3 °C, 1 min at 72 °C for 35 cycles, 10 min at 72 °C |
| AD5RO/AAP | 10 min at 94 °C, 40 s at 94 °C, 40 s at 51.3 °C, 1 min at 72 °C for 35 cycles, 10 min at 72 °C |
| AD5RI/AUAP | 10 min at 94 °C, 40 s at 94 °C, 40 s at 55.3 °C, 1 min at 72 °C for 35 cycles, 10 min at 72 °C |
| ADPETF/ADPETR | 10 min at 94 °C, 40 s at 94 °C, 40 s at 61.6 °C, 1 min 20 s at 72 °C for 35 cycles, 10 min at 72 °C |
| ADPF/ADPR | 10 min at 94 °C, 40 s at 94 °C, 40 s at 51 °C, 1 min 30 s at 72 °C for 35 cycles, 10 min at 72 °C |
| G1/G2 | 10 min at 94 °C, 40 s at 94 °C, 40 s at 51.8 °C, 1 min at 72 °C for 35 cycles, 10 min at 72 °C |
| G3/G4 | 10 min at 94 °C, 40 s at 94 °C, 40 s at 47 °C, 1 min at 72 °C for 35 cycles, 10 min at 72 °C |
| G5/G6 | 10 min at 94 °C, 40 s at 94 °C, 40 s at 49 °C, 1 min at 72 °C for 35 cycles, 10 min at 72 °C |
| G7/G8 | 10 min at 94 °C, 40 s at 94 °C, 40 s at 48 °C, 1 min at 72 °C for 35 cycles, 10 min at 72 °C |
