# Supplementary material for: Characterization of a Decapentapletic Gene (AccDpp) from Apis cerana cerana and Its Possible Involvement in Development and Response to Oxidative Stress
Source: PLoS One. 2016 Feb 16;11(2):e0149117. doi: 10.1371/journal.pone.0149117 (PMC4755538; doi:10.1371/journal.pone.0149117)
Supplement: S4 Table — (DOC) [file pone.0149117.s005.doc]

**S4 Table.** Characterisation of gene used in this paper.

| Source of species | Gene name | Genbank accession number |
| --- | --- | --- |
| *Apis cerana cerana* | *AccDpp* | KT750952 |
| *Bombus terrestris* | *BtDpp-like* | XP_003401984.1 |
| *Megachile rotundata* | *MrDpp-isoform-X1* | XP_012143496.1 |
| *Apis mellifera* | *AmDpp* | XP_006569849.1 |
| *Atta cephalotes* | *AcDpp* | XP_012063732.1 |
| *Camponotus floridanus* | *CfDpp-isoform-X1* | XP_011257742.1 |
| *Pogonomyrmex barbatus* | *PbDpp* | XP_011637871.1 |
| *Athalia rosae* | *ArDpp* | NP_001295470.1 |
| *Orussus abietinus* | *OaDpp* | XP_012281174.1 |
| *Diaphorina citri* | *DcDPP* | XP_008483895.1 |
| *Drosophila melanogaster* | *DmDpp* | gb|AAA28482.1 |
| *Octodon degus* | *OdBMP4* | XP_004638796.1 |
| *Trichechus manatus latirostris* | *TmlBMP2* | XP_004383108.1 |
| *Zonotrichia albicollis* | *ZaBMP4* | XP_005495258.1 |
| *Mesocricetus auratus* | *MaBMP2* | XP_005068733.1 |
| *Lasius niger* | *LnDpp* | KMQ95783.1 |
| *Tribolium castaneum* | *TcDpp* | NP_001034540.1 |
| *Branchiostoma floridae* | *BfBMP2/4* | AAC97488.1 |
