# Supplementary material for: Characterization of a Decapentapletic Gene (AccDpp) from Apis cerana cerana and Its Possible Involvement in Development and Response to Oxidative Stress
Source: PLoS One. 2016 Feb 16;11(2):e0149117. doi: 10.1371/journal.pone.0149117 (PMC4755538; doi:10.1371/journal.pone.0149117)
Supplement: S5 Table — (DOC) [file pone.0149117.s006.doc]

**S5 Table.** The basic subunit of AccDpp secondary structure.

| Amino acid | Subunit | Amino acid | Subunit |
| --- | --- | --- | --- |
| 17-34 | α_1_ | 226-231 | β_9_ |
| 50-59 | α_2_ | 240-247 | α_4_ |
| 79-83 | β_1_ | 255-259 | α_5_ |
| 100-104 | β_2_ | 267-273 | β_10_ |
| 117-122 | β_3_ | 290-296 | β_11_ |
| 138-146 | β_4_ | 313-317 | α_6_ |
| 148-151 | β_5_ | 333-338 | β_12_ |
| 154-166 | β_6_ | 340-344 | β_13_ |
| 174-178 | β_7_ | 352-356 | β_14_ |
| 179-186 | α_3_ | 360-365 | β_15_ |
| 193-200 | β_8_ |  |  |
