# Supplementary material for: Characterization of a Decapentapletic Gene (AccDpp) from Apis cerana cerana and Its Possible Involvement in Development and Response to Oxidative Stress
Source: PLoS One. 2016 Feb 16;11(2):e0149117. doi: 10.1371/journal.pone.0149117 (PMC4755538; doi:10.1371/journal.pone.0149117)
Supplement: S6 Table — (DOC) [file pone.0149117.s007.doc]

**S6 Table.** Exons and introns size and GC content of the deosited *Dpp* gene in NCBI.

| Exon on. | Gene | Length  (bp) | GC content (%) | Intron on. | Gene | Length  (bp) | GC content (%) |
| --- | --- | --- | --- | --- | --- | --- | --- |
| 1 | *AccDpp* | 328 | 40.85% | 1 | *AccDpp* | 1954 | 15.10% |
|  | *AmDpp* | 306 | 40.20% |  | *AmDpp* | 2005 | 14.91% |
|  | *BtDpp-like* | 328 | 42.38% |  | *BtDpp-like* | 4066 | 25.93% |
|  | *MrDpp-isoform-X1* | 328 | 43.29% |  | *MrDpp-isoform-X1* | 2847 | 25.64% |
|  | *AcDpp-like* | 343 | 58.89% |  | *AcDpp-like* | 5559 | 26.65% |
|  | *DcDpp* | 304 | 44.08% |  | *DcDpp* | 566 | 36.75% |
| 2 | *AccDpp* | 445 | 33.48% | 2 | *AccDpp* | 285 | 10.88% |
|  | *AmDpp* | 461 | 32.32% |  | *AmDpp* | 186 | 9.14% |
|  | *BtDpp-like* | 445 | 37.75% |  | *BtDpp-like* | 310 | 17.74% |
|  | *MrDpp-isoform-X1* | 442 | 36.20% |  | *MrDpp-isoform-X1* | 134 | 19.40% |
|  | *AcDpp-like* | 860 | 55.81% |  | *DcDpp* | 89 | 35.96% |
|  | *DcDpp* | 541 | 40.67% | 3 | *AccDpp* | 175 | 8.57% |
| 3 | *AccDpp* | 400 | 38.25% |  | *AmDpp* | 127 | 11.02% |
|  | *AmDpp* | 400 | 38.75% |  | *BtDpp-like* | 370 | 16.76% |
|  | *BtDpp-like* | 400 | 40.75% |  | *MrDpp-isoform-X1* | 119 | 16.81 |
|  | *MrDpp-isoform-X1* | 400 | 40.50% |  |  |  |  |
|  | *DcDpp* | 337 | 44.51% |  |  |  |  |
